# CSNet: A Count-Supervised Network via Multiscale MLP-Mixer for Wheat Ear Counting

**DOI:** 10.34133/plantphenomics.0236

**Published:** 2024-08-20

**Authors:** Yaoxi Li, Xingcai Wu, Qi Wang, Zhixun Pei, Kejun Zhao, Panfeng Chen, Gefei Hao

**Affiliations:** ^1^ State Key Laboratory of Public Big Data, College of Computer Science and Technology, Guizhou University, Guiyang 550025, China.; ^2^ Department of Computer Science and Technology, Tsinghua University, Beijing 100084, China.; ^3^ National Key Laboratory of Green Pesticide, Key Laboratory of Green Pesticide and Agricultural Bioengineering, Ministry of Education, Guiyang 550025, China.

## Abstract

Wheat is the most widely grown crop in the world, and its yield is closely related to global food security. The number of ears is important for wheat breeding and yield estimation. Therefore, automated wheat ear counting techniques are essential for breeding high-yield varieties and increasing grain yield. However, all existing methods require position-level annotation for training, implying that a large amount of labor is required for annotation, limiting the application and development of deep learning technology in the agricultural field. To address this problem, we propose a count-supervised multiscale perceptive wheat counting network (CSNet, count-supervised network), which aims to achieve accurate counting of wheat ears using quantity information. In particular, in the absence of location information, CSNet adopts MLP-Mixer to construct a multiscale perception module with a global receptive field that implements the learning of small target attention maps between wheat ear features. We conduct comparative experiments on a publicly available global wheat head detection dataset, showing that the proposed count-supervised strategy outperforms existing position-supervised methods in terms of mean absolute error (MAE) and root mean square error (RMSE). This superior performance indicates that the proposed approach has a positive impact on improving ear counts and reducing labeling costs, demonstrating its great potential for agricultural counting tasks. The code is available at http://csnet.samlab.cn.

## Introduction

Automated technology is vital for wheat food security, because it enhances breeding efficiency and food production. Wheat is one of the most important food crops, providing approximately 20% of the world’s protein and carbohydrate intake and bearing the burden of global food security [1]. In addition, wheat has various uses, including industrial raw material, biofuel, and animal feed. However, as the global population continues to grow and the world develops, the growth in wheat production has not matched that of demand. An in-depth analysis reveals that the annual growth rate of wheat demand is 1.7%, juxtaposed with a modest average annual rate of genetic increase of only 1% [2]. Therefore, automation technology has been fully utilized to improve breeding efficiency and cope with the global food crisis. More specifically, automation technology can use modern computers to replace manual statistical analysis of crop phenotypes (including height, color, number of ears, and other relevant phenotypes) [3], thus reducing labor and time costs and achieving efficient breeding.

Selecting varieties with desirable traits through high-quality automated counting is an essential process in breeding. Wheat yield, one of the most important traits, is determined by 3 elements: the number of wheat ears per unit ground area, number of grains, and weight of 1,000 grains [4]. Conventional breeding methods rely on manual counting to ascertain the number of wheat ears, a process that is prone to low efficiency, high time and cost, and high error [5]. Consequently, the implementation of automated counting is indispensable for enhancing breeding efficiency and conserving human resources. To achieve high-quality and automated counting, researchers have begun to explore the potential of image-processing techniques for recognizing wheat ears. For instance, Cointault et al. [6] used color and texture feature processing techniques to achieve wheat ear segmentation in images. Alharbi et al. [7] utilized a Gabor filter and the K-means clustering algorithm to detect segmented wheat ear regions and perform wheat ear counting. Fernandez et al. [8] proposed a high-throughput and low-cost method for wheat ear counting by utilizing Laplace frequency and median filters to obtain low-noise wheat ear features. However, the aforementioned methods have mediocre generalization ability and are easily affected by interference factors, such as illumination and environment, limiting their suitability for wheat ear images with rich backgrounds and diverse morphologies.

With the rapid development of deep learning, position-supervised methods, including box-supervised and point-supervised wheat counting approaches, have garnered substantial attention. On the one hand, bounding boxes are employed to select and quantify the number of wheat ears in terms of box-supervised wheat ear counting methods. For instance, Li et al. [9] utilized a Faster R-CNN trained on a self-constructed dataset to achieve fast recognition of wheat ears. Gong et al. [10] proposed a 2-space pyramid pooling network to improve YOLOv4, which further enhanced the detection accuracy of wheat ears. Zang et al. [11] improved the YOLOv5s model for detecting small-scale wheat ear counts. On the other hand, predicting the density map of wheat ears can achieve counting in the case of point-supervised wheat ear counting methods. Lu et al. [12] proposed a local counting regression network known as TasselNet to address the problem of counting maize tassels in the wild. Xiong et al. [13] improved the accuracy and efficiency of wheat ear counting by adding contextual information to TasselNet. Khaki et al. [14] designed a lightweight wheat ear counting model with MobileNetV2 as the backbone, relying on a density map for the counting and localization of wheat ears. Ma et al. [15] selected filtered pyramid blocks and dilated convolutions to construct EarDensityNet, which predicts wheat ear density maps to obtain the number of ears. Wu et al. [16] constructed and optimized a density graph regression network for wheat ear counting in unmanned aerial vehicle (UAV) images. The aforementioned methods address the problems of mediocre generalization and susceptibility to noise and have achieved excellent results in wheat ear counting; however, they require training on high-cost position-level images.

**Fig. 1.**
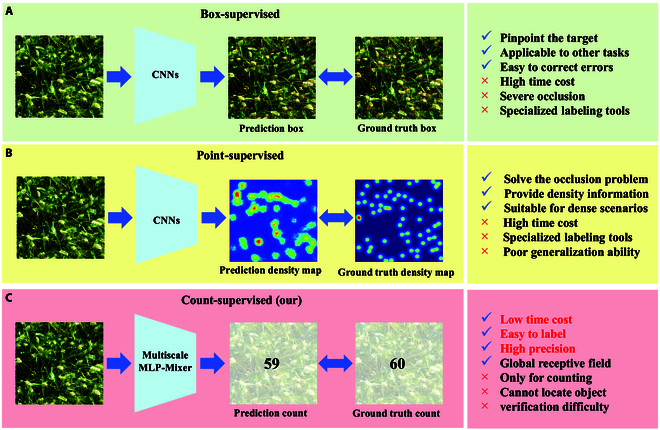
(A) Box-supervised CNN-based methods, which can predict the target box to locate the wheat ear, but are costly to label and are poor at dealing with the overlapping wheat ear. (B) Point-supervised CNN-based methods, which predict the density map to obtain the number of wheat ears, are costly to label and are poorly suited to wheat ears of varying lengths. (C) The proposed CSNet is a multiscale global perception method based on count-supervised, which is easy to label, low cost, and highly accurate.

Both box-supervised and density map-based point-supervised wheat ear counting models are locally perceptive convolutional neural networks (CNNs) [17] that can use location information (boxes or density maps) to obtain wheat ear features, as shown in Fig. 1. However, the dense and varied location information of wheat ears is not only costly to label but also introduces inevitable noise that may distract the attention of the model to the wheat ears, resulting in a limitation of model performance. In particular, box-supervised wheat ear counting methods use several target boxes to locate wheat ears and remove duplicate boxes using a nonmaximum suppression technique [18]; however, this is not sufficiently accurate for overlapping wheat ears. In point-supervised methods focusing on dense targets that use Gaussian kernels of the same size to achieve wheat ear localization, adaptation to wheat ears of varying lengths is difficult [19]. Furthermore, the application of the Gaussian kernel to generate density maps inevitably results in the labeling of the surrounding backgrounds of wheat ears with densities, thereby introducing background noise. Therefore, a low labeling cost and location-independent method for counting wheat ears is crucial for increasing wheat yield.

In the field of crowd counting, researchers have recently explored count-supervised methods to reduce annotation costs and enhance counting accuracy. For instance, Yang et al. [20] proposed a soft-label sorting and counting networks that achieves count-supervised crowd counting. Liang et al. [21] presented TransCrowd, a crowd-counting network based on a transformer [22] architecture, that effectively extracts semantic crowd information through a self-attention mechanism. Wang et al. [23] presented a multi-granularity multilayer perceptron (MLP) to mine global information and overcome the lack of spatial cues through a proxy task known as split counting. The aforementioned methods are primarily designed to address the challenges posed by considerable density variations within crowds, making them less suitable for wheat ear scenarios characterized by smaller density variations. To address the challenges encountered in wheat ear scenarios within count-supervised, we propose a novel count-supervised wheat counting network known as CSNet, which is a multiscale global perception model for achieving accurate and efficient wheat ear counting with count information only. Specifically, we design a multiscale perception module (MPM) based on the MLP-Mixer network [24] with a global perception capability to learn wheat ear features from different spatial dimensions. For dense or differently sized wheat ears, the MLP-Mixer constructs global feature relationships to obtain the attention map of wheat ears without complex labeling information. Furthermore, we introduce a convolutional block attention module (CBAM) [25] to reduce the effects of background information. In the experiments, we validate the proposed CSNet on a global wheat head detection (GWHD) [26,27] dataset, achieving state-of-the-art results compared with the location-supervised advanced approach. Regarding dataset usage, the proposed CSNet uses labeled data at a much lower cost than location-supervised methods, exhibiting considerable potential for agricultural counting. In summary, the main contributions of this paper are as follows:

• To the best of our knowledge, this is the first study to propose a count-supervised wheat counting method that yields high-precision results at low labeling costs.

• We design an MPM that obtains attention maps of wheat ears in different spatial dimensions by constructing global feature relations, enabling the model to effectively handle diverse wheat ear sizes while relying solely on count information.

• We conduct quantitative and qualitative experiments on the GWHD dataset, manifesting the effectiveness of the CSNet and generalizability of similar agricultural counting tasks.

## Materials and Methods

In this section, we introduce the multiscale count-supervised network (CSNet). The “Dataset” section describes the dataset required for the experiment. In the “Methods” section, we describe the framework of CSNet. The “Evaluation metrics” section introduces the evaluation metrics used in the counting model.

### Dataset

**Fig. 2.**
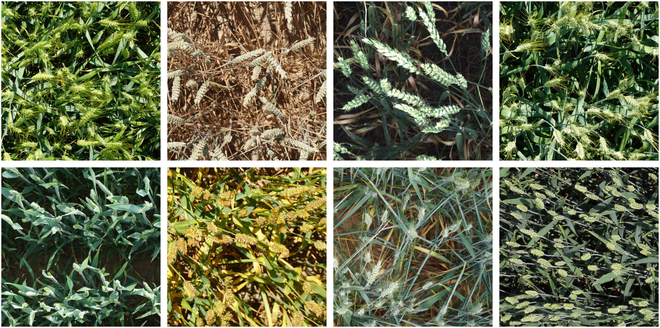
Image diversity examples in GWHD_2021 dataset.

The range of wheat cultivation is unrivaled, and it is grown in almost every country [28]. Wheat varieties vary across different regions because of different natural conditions such as climate, soil, and light. Consequently, creating a universal wheat ear dataset remains challenging. To address this issue, David et al. [26] proposed the GWHD_2020 dataset, which is the first publicly available dataset of wheat crops from multiple countries. In particular, the GWHD_2020 dataset contains wheat varieties at different growth stages and a wide range of genotypes from Europe, North America, Australia, and Asia, totaling 4,700 RGB images containing 193,634 labeled wheat ears. Among them, the image data in the GWHD_2020 dataset were collected at heights ranging from 1.8 to 3 m above the ground, and data harmonization was performed after collection to ensure that all images in the dataset were clearly visible [26]. As shown in Fig. 2, the GWHD_2020 dataset contains a wide range of growth stages and varieties of wheat that vary in color, shape, size, and tilt angle. In 2021, the GWHD_2020 dataset was expanded and updated with the addition of 1,722 images and 81,553 labeled wheat ears from 5 additional countries, making it a larger, more diverse, and less noisy dataset [27], which we refer to as GWHD_2021 dataset. They have a substantial impact on wheat counting and provide invaluable resources for innovative research and advancement in wheat-related studies.

We selected the aforementioned dataset for the experiments to verify the validity of the proposed model. As summarized in Table 1, we used 3,422 images from the GWHD_2020 dataset for the experiments, where the maximum number of wheat ears in a single image was 112, minimum value was 0, and average value was 42.49, totaling 145,411 wheat ears. In terms of the GWHD_2021 dataset, we exploited 6,509 images for experiments, with a range of wheat ear counts per image of 0 to 190, averaging 42.29 ears per image and totaling 275,260 ears in the dataset. Based on the common method of dataset division, we randomly selected 80% of the dataset as training data, 10% as validation data, and 10% as test data.

**Table 1.** The statistics of the dataset used in this study. Min, Max, Avg, and Total denote the minimum, maximum, average, and total number of annotated wheat ears, respectively.

Dataset	Subset	Number of images	Min	Max	Avg	Total
GWHD (2020) [26]	Train	2,738	0	112	42.49	116,350
	Val	342	0	88	44.58	15,245
	Test	342	0	94	40.40	13,816
GWHD (2021) [27]	Train	5,206	0	190	42.41	220,796
	Val	651	0	146	41.13	26,779
	Test	652	0	159	42.46	27,685

In addition, we constructed a self-contained wheat grain dataset containing 510 images as an extended test. In particular, the number of wheat grains in the images ranged from 0 to 68, with an average of 38.9 grains per image and 19,839 grains. Similarly, we randomly divided 80% of the data into training data and the remainder into test data.

### Methods

**Fig. 3.**
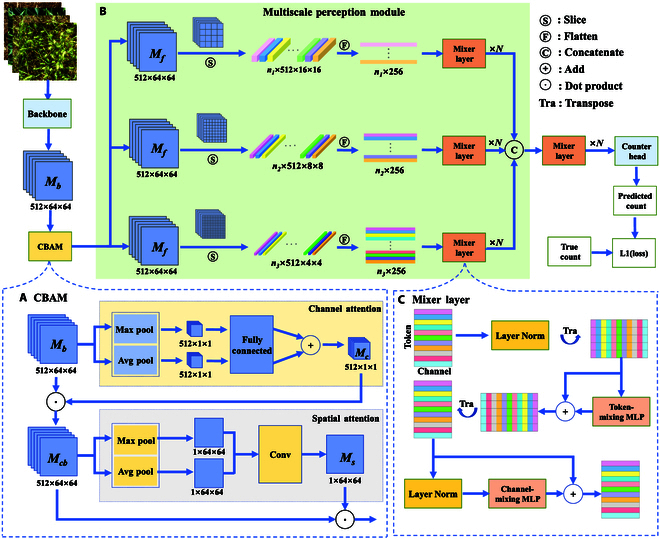
The overall architecture of CSNet, including Backbone, convolutional block attention module (CBAM), multiscale perception module (MPM), and counting module (CM). (A) The detailed structure of CBAM is used to improve the attention to the wheat region. (B) The flow of the MPM utilizes the wheat ear features at multiple scales to improve the generalization performance of the network. (C) The detailed operation of the mixer layer in the MPM achieves sensing wheat ears without location information by fusing global features.

In this study, we propose a novel count-supervised wheat ear counting network known as CSNet, which comprises a backbone, CBAM, MPM, and a counting module (CM), as shown in Fig. 3. In particular, the backbone extracts image features, whereas the CBAM focuses on wheat region features. To further adapt to the diversity of wheat ears, we design an MPM to obtain the features of wheat ears in multiple spatial dimensions, which improves the ability of the model to recognize wheat ears. Finally, the CM uses a fully connected layer and an average pooling layer to directly regress the final counting results. In the following subsections, we elaborate on the implementation principles for each part.

#### Backbone

The backbone is an important component of the neural network because it is responsible for feature extraction and has a substantial impact on the generalization ability, robustness, and overall efficiency of the model [29]. To optimize the balance of the model between accuracy and resource overhead, we selected the first 10 layers of VGG16 [30], including the first ten 3 × 3 convolutional layers and 3 max-pooling layers, as the backbone of CSNet [31]. This backbone is pretrained on ImageNet and initially has the ability to extract the underlying features, which results in notable advantages such as saving computational resources, increasing computational efficiency, and improving the generalization ability of the model.

#### Convolutional block attention module

In the context of a complex environment with weeds that are overgrown and wheat that is obscured from each other, the model needs to focus its limited attention on the area with wheat ears to be able to count the ears effectively. To address this problem, we introduce an efficient and lightweight CBAM [25] that combines channel and spatial attention. In particular, channel attention can adjust the attention degree of the model between each feature to focus on essential features (e.g., shape, size, and texture of the wheat ears) and ignore irrelevant features (e.g., light variations and debris on the wheat ears). Spatial attention adjusts the extent to which the model focuses on each region of the image, thereby enhancing attention to the wheat region and reducing the influence of background regions (e.g., weeds).

As shown in Fig. 3, the backbone layer outputs feature map *M_b_*, which is further optimized using the CBAM module to obtain feature map *M_f_*. Compared with feature map *M_b_*, feature map *M_f_* focuses more on wheat ears in both the channel and space. Initially, the spatial average and max pooling operations are executed on *M_b_* to derive the maximum and average values for each channel, respectively. Subsequently, the maximum and average of each channel are weighted through the fully connected layer to obtain the channel attention weight, expressed as *M_s_*, reflecting the degree of attention assigned to a single channel. In addition, the channel attention feature map, denoted by *M_cb_*, is obtained by performing a point similarity operation on the channel attention weight *M_c_* and feature map *Mb*. Subsequently, channel average and max pooling are performed on *M_cb_*, and the results are passed through a convolutional layer to obtain attention weight *M_s_*, which contains the spatial location information. Ultimately, the dot multiplication of each spatial location in *M_cb_* with attention weights *M_s_* produces a feature map, denoted as *M_f_*, which is augmented with attention to the wheat ears in both the channel and space. This process enhances the perceptual focus on the wheat region and emphasizes the crucial features of the wheat ear. The CBAM process is formally expressed as follows:

$$\begin{array}{c} M_{c}=\sigma \left(\text{FC}\left({\text{pool}}_{\max}^{c}\left(M_{b}\right)\right)+\text{FC}\left({\text{pool}}_{\mathrm{avg}}^{c}\left(M_{b}\right)\right)\right), \end{array}$$ (1)

$$M_{\mathrm{cb}}=M_{c}\times M_{b},$$ (2)

$$\begin{array}{c} M_{s}=\sigma \left(\text{conv}\left({\text{pool}}_{\text{max}}^{s}\left(M_{\mathrm{cb}}\right)+{\text{pool}}_{\text{avg}}^{s}\left(M_{cb}\right)\right)\right), \end{array}$$ (3)

$$M_{f}=M_{s}\times M_{cb},$$ (4)

where *σ* denotes a sigmoid function, *FC* represents a fully connected layer, ${\text{pool}}_{\text{max}}^{c}$ denotes the spatial max pooling, and ${\text{pool}}_{\text{avg}}^{s}$ denotes the average channel pooling.

#### Multiscale perception module

Both box-supervised [9–11] and point-superved [14–16] rely on positional information to recognize diverse and dense wheat ears; however, annotating and overlapping wheat ears is costly [32], and the subjectivity of the labeler can lead to ambiguity. Therefore, we believe that location information may not be essential for wheat ear counting, leading to the design of the MPM to perceive diverse and dense wheat ears using only quantity information.

To perceive wheat ears in the absence of positional information, we adopt the mixer layer of the MLP-Mixer [24] network, which is based on MLP, to learn the relationship between each patch and all other patches, thus sensing the connection between wheat ears and counting. However, the phenotypes (size, color, and shape) of wheat ears are so diverse that perceiving all wheat ears on a single scale is impossible. To address this problem, we propose a multiscale method that captures wheat ear features in multiple spaces for accurate recognition. As shown in Fig. 3, the feature maps are sliced into patches of different sizes, with smaller patches capturing more subtle features. By perceiving features at different scales, the MPM can distinguish features from multiple spatial dimensions to identify diverse wheat ears.

In detail, the MPM mainly slices and projects the wheat ear feature map *M_f_* onto multiple feature matrices, which are created to obtain comprehensive global attention information using the mixer layer for information interaction, as shown in Fig. 3. First, the wheat ear feature map *M_f_* output from CBAM is sliced into feature patches of *n*_1_ × 512 × 16 × 16, *n*_2_ × 512 × 8 × 8, and *n*_3_ × 512 × 4 × 4 sizes, *n*_1_, *n*_2_, and *n*_3_ corresponding to the values of 16, 64, and 256, respectively. The smaller the slice size, the larger the number of patches. Each feature patch is then mapped as a feature vector, thus constituting a feature matrix in which the same rows in the feature matrix represent different channels in the same space, and the same columns represent the same channels in different spaces. Furthermore, the feature matrix is fed into the mixer layer for information interaction, which comprises Layer Norm and an MLP, and each row of the feature matrix is normalized by Layer Norm and then communicated through multi-MLPs. In addition, the rows of feature matrices, representing different spatial or channel information, undergo reciprocal transformations via transposition and engage in MLPs to obtain comprehensive global attention information. Finally, the MPM concatenates the 3 feature matrices with different scale information and interacts with them again through the mixer layer, producing a wheat ear feature matrix that incorporates global attention into 3 dimensions. The mixer layer fuses and optimizes features from multiple scales, eliminating discrepancies and promoting a consistent feature representation. The described feature matrices are denoted by *T*_1_, *T*_2_, *T*_3_, and *T_all_*, and the entire process can be defined as follows:

$$T_{1}={\text{Mix}}_{1}^{N}\left(F_{1}\left(S_{16\times 16}\left(M_{f}\right)\right)\right.,$$ (5)

$$T_{2}={\text{Mix}}_{2}^{N}\left(F_{2}\left(S_{8\times 8}\left(M_{f}\right)\right)\right.,$$ (6)

$$T_{3}={\text{Mix}}_{3}^{N}\left(F_{3}\left(S_{4\times 4}\left(M_{f}\right)\right)\right.,$$ (7)

$$T_{\text{all}}={\text{Mix}}_{\text{all}}^{N}\left(\text{Concat}\left(T1,T2,T3\right)\right),$$ (8)

where *S* denotes the slice operations, 16 × 16 denotes the size of the sliced patch, and *F_i_* denotes a linear projection. In addition, ${\text{Mix}}_{i}^{N}$ indicates that *N* mixer layers exist on the *i*th scale.

#### Counting module

The CM is designed to convert features into quantities without the need to generate bounding boxes or density maps, but rather to generate regression counts directly. In particular, the proposed CM uses the information-rich wheat ear feature matrix output from the MPM input to the fully connected layer for dimensionality reduction and generates counts. To mitigate the potential for considerable discrepancies owing to inherent variability in individual counts, the CM concurrently predicts a set of counts and subsequently aggregates the final predicted number of wheat ears via average pooling. The detailed process is as follows:

$$x_{1}=\sigma \left({\text{FC}}_{1}\left(T_{\text{all}}\right)\right),$$ (9)

$$x_{2}={\text{FC}}_{2}\left(\text{Dropout}\left(x_{1}\right)\right),$$ (10)

$$\hat{C}=\sigma \left({\text{pool}}_{\text{avg}}\left(x_{2}\right)\right),$$ (11)

where *σ* denotes the *ReLU* function and $\hat{C}$ denotes the final predicted count.

### Evaluation metrics

To investigate the counting performance of the model, we utilize the mean absolute error (MAE), root mean square error (RMSE), and *R*-squared metrics to evaluate the performance in the counting task. MAE is the difference between the predicted and actual values, and it is used to assess the accuracy of the model. The RMSE is the deviation between the predicted and true values, which is used to measure the stability of the model. *R*^2^ is a statistic used to measure the extent to which the regression model fits the data and can take a range of values from 0 to 1; the closer it is to 1, the better the regression model fits the data. The formulas for the above evaluation metrics are expressed as follows:

$$\mathrm{MAE}=\frac{1}{N}\sum_{i=1}^{N}‍\left|{\hat{C}}_{i}-C_{i}\right|,$$ (12)

$$\text{RMSE}=\sqrt{\frac{1}{N}\sum_{i=1}^{N}‍{\left({\hat{C}}_{i}-C_{i}\right)}^{2}},$$ (13)

$$R^{2}=1-\frac{\sum_{i=1}^{N}‍{\left({\hat{C}}_{i}-C_{i}\right)}^{2}}{\sum_{i=1}^{N}‍{\left(\bar{C}-C_{i}\right)}^{2}},$$ (14)

where ${\hat{C}}_{i}$ denotes the estimated total number of wheat ears in the *i*th image, *C_i_* denotes the real number in the *i*th image, $\bar{C}$ denotes the average real number, and *N* denotes the number of predicted images.

## Results

### Experimental details

CSNet is optimized using the stochastic gradient descent (SGD) algorithm, and the training batches are set to 16. We use the MultiStepLR scheduler to adjust the learning rate with an initial learning rate of 1 × 10^−4^ and employ the L1 loss function as the loss criterion. Compared with the L2 loss function, the L1 loss function is less affected by challenging scenarios and prevents the model from being overly influenced by outliers. To accommodate multiscale slicing, all images are uniformly scaled to a size of 512 ×512. In addition, the number *N* of the mixer layers is fixed at 4. All experiments are implemented in the PyTorch framework and uniformly trained on NVIDIA A40 GPU for approximately 40 h using the original configuration, and the weights of the experiment that worked best on the validation dataset are taken for testing. Besides, we evaluate the performance of the proposed method: 4 methods based on box-supervised, 5 based on point-supervised, and 1 based on count-supervised on 2 datasets.

### Performance comparison

To validate the effectiveness of the proposed CSNet, we compare the proposed method with box-supervised and point-supervised methods commonly used for wheat counting and count-supervised methods used for crowds on the GWHD_2020 and GWHD_2021 datasets, as summarized in Table 2. Among them, box-supervised methods include single-stage target detection methods SSD [33] and YOLOv8 [34], a 2-stage target detection method Faster R-CNN [35], and a transform-based target detection method DETR [36]. For the point-supervised methods, we conduct experiments using MCNN [37], CSRNet [31], ASD [38], SPN [39], and WheatNet [14]. Furthermore, we compare the proposed method with the count-supervised method used for crowds known as TransCrowd [21].

**Table 2.** Performance comparison of competing methods using different supervision on the 2020 version of the GWHD dataset and the latest 2021 version

Method (years)	Supervision	GWHD_2020		GWHD_2021	
		MAE	RMSE	MAE	RMSE
Faster R-CNN (2015) [35]	Box	3.27	4.49	5.46	9.76
SSD (2016) [33]	Box	3.97	5.44	7.19	12.08
DETR (2022) [36]	Box	3.76	4.91	8.41	16.20
YOLOv8 (2023) [34]	Box	4.39	6.17	8.96	13.43
MCNN (2016) [37]	Point	5.05	6.47	7.59	10.17
CSRNet (2018) [31]	Point	3.87	5.01	5.88	8.13
ASD (2019) [38]	Point	5.86	7.55	/	/
SPN (2019) [39]	Point	5.91	7.73	/	/
WheatNet (2022) [14]	Point	3.85	5.19	/	/
TransCrowd-Token (2022) [21]	Count	15.87	19.02	11.29	14.39
TransCrowd-GAP (2022) [21]	Count	12.23	15.02	10.09	12.85
CSNet (ours)	Count	2.94	3.88	3.87	5.60

As summarized in Table 2, the proposed CSNet outperforms both the box-supervised and point-supervised methods in terms of MAE and RMSE. In the GWHD_2020 dataset, the MAE of CSNet is 10.1% lower than that of the best box-supervised model (Faster R-CNN) and 23.6% lower than that of the best point-supervised model (CSRNet), which shows that the proposed count-supervised model can achieve better performance in the absence of location information. Comparing the 2 location-supervised methods, the box-supervised model outperforms the point-supervised model on average, exhibiting the best MAE of 3.27 and 3.85, and the worst MAE of 4.39 and 5.91, respectively. The MAE of the best model in the box-supervised approach is reduced by 25.5% compared to that of the worst model, whereas it is reduced by 34.8% in the point-supervised approach, demonstrating that a considerable difference exists between the models using the same methods.

Notably, the inclusion of dense images in the GWHD_2021 dataset resulted in a decrease in the performance of all models. Compared with the GWHD_2020 dataset, the MAE of CSNet improves by 31.6%, that of Faster R-CNN improves by 66.9%, and that of CSRNet improves by 51.9%. CSNet continues to exhibit excellent performance on the GWHD_2021 dataset, which may depend on the fact that it is not limited by location information, thereby maximizing its perceptual ability. The substantial increase in the RMSE evaluation metrics of the box-supervised methods (e.g., that of DETR is as high as 16.2) reveals their disappointing prediction of dense images, which is the main reason for the considerable increase in their MAE. The overlapping target boxes in dense scenes considerably affect the performance of the box-supervised method.

TransCrowd [21] is a count-supervised model with excellent crowd-counting performance that can be classified into 2 modes, token and GAP, depending on the final output type. However, it exhibits a less satisfactory performance in the wheat counting task because the wheat scene was notably different from the crowd scene. As summarized in Table 2, although the GAP mode of TransCrowd outperforms that of the Token mode, it still falls short compared to all the methods we experimented with. We attribute this underperformance to the substantial differences between the wheat and crowd scenes, making them unsuitable for wheat ear counting.

**Fig. 4.**
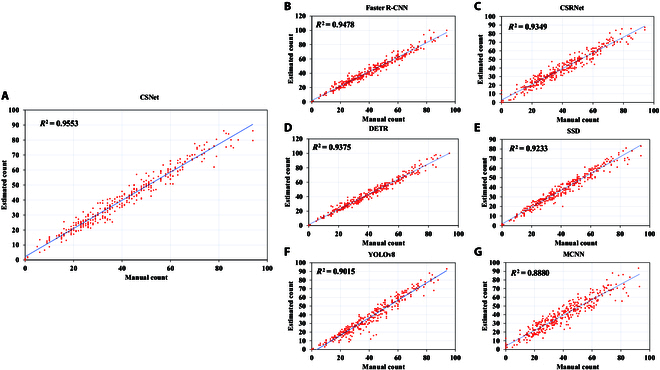
Coefficient of determination of the model on the GWHD_2020 dataset. (A), (B), (C), (D), (E), (F), and (G) represent CSNet, Faster R-CNN, CSRNet, DETR, SSD, YOLOv8, and MCNN models, respectively.

To further explore the performance of the models, we use the *R*^2^ metric to determine the degree of linear regression straight line fit of each model on the GWHD_2020 dataset. As shown in Fig. 4, the *R*^2^ metric of CSNet reaches 0.95, which is higher than that of the other models, proving that CSNet can effectively fit the number of ears without relying on location information. Clearly, the proposed method can obtain excellent results with reduced labeling, which is of great importance for decreasing the application cost of counting models and promoting the development of counting tasks in agriculture.

### Impact of different backbones

Classical networks are selected as the backbone of CSNet, including VGG16 [30], ResNet34 [40], ResNet50 [40], MobileNetV2 [41], DarkNet53 [42], and ViT [43], to further evaluate the impact of the backbone on the model performance. All models are pretrained on ImageNet to learn the generic raw features. As summarized in Table 3, using VGG16 as the backbone enables the proposed model to achieve the best counting accuracy on the GWHD_2020 and GWHD_2021 datasets, which may be attributed to the fact that only the features of a single object (wheat ear) need to be captured in the wheat ear counting task without the need to use a more complex network structure. The counting accuracies of ResNet50 and DartNet53, which have more parameters than VGG16, do not increase but decrease. This may be because the wheat counting task is simple and only needs to focus on the features of wheat ears; therefore, the backbone with a large number of parameters appears to be overfitted. However, the transform-based model ViT does not perform well as a backbone, most likely because it requires a large amount of data to exploit its performance. Furthermore, when employing the lightweight MobileNetV2 backbone, CSNet exhibits commendable performance on the GWHD_2020 dataset, but achieves suboptimal results on the GWHD_2021 dataset. This suggests that MobileNetV2 is better suited for simpler scenarios, providing an ideal solution for environments in which inference speed is a priority in uncomplicated settings. CSNet also exhibits good performance when using lightweight Mobilenetv2 as the backbone, which provides a well-suited solution for environments where the speed of inference is sought. In conclusion, we observe that the backbone network has a considerable impact on model performance, and selecting the appropriate network for the task can notably affect the counting accuracy and generalization ability of the model.

**Table 3.** Counting performance of our proposed method under different backbones on the GWHD dataset

Backbone	GWHD_2020		GWHD_2021		Number of parameters (M)
	MAE	RMSE	MAE	RMSE	
VGG16	2.94	3.88	3.87	5.60	7.6
MobileNetV2	3.47	4.55	7.52	10.24	0.6
ResNet34	4.02	5.22	5.94	8.22	21.3
ResNet50	4.62	6.05	6.33	9.2	25.5
DartNet53	3.83	5.09	4.97	7.68	40.6
ViT	9.36	12.24	14.51	18.95	21.7

### Impact of the CBAM

To explore the effects of CBAM on the attentional mechanisms of the model, we conduct a series of experiments to compare the model performance with and without CBAM. As summarized in Table 4, we not only compare their overall performance on the entire set of test data but also perform a detailed examination of their counting capabilities in more densely populated scenes with counts greater than 40 and less dense scenarios with counts less than 40. When CBAM is utilized, a noticeable reduction in both the MAE and RMSE is present compared to the variant without CBAM in denser scenarios. More specifically, in the GWHD_2020 dataset, although the MAE of the model with CBAM is slightly higher than that of the model without CBAM in less dense scenarios, its lower RMSE indicates that the CBAM-enhanced model has better attention generalization and accuracy. This suggests that CBAM plays a crucial role in refining the attention mechanism of the model, allowing it to adapt and excel in challenging and dense wheat ear configurations.

**Table 4.** Counting performance with and without CBAM on the GWHD_2020 and GWHD_2021 datasets

Dataset	CBAM	MAE	RMSE	MAE_≤40_	RMSE_≤40_	MAE_>40_	RMSE_>40_
2020		3.20	4.36	2.59	3.47	3.89	5.17
	✓	2.94	3.88	2.63	3.37	3.31	4.38
2021		4.13	5.94	3.11	4.10	5.24	7.45
	✓	3.87	5.60	2.90	3.76	4.93	7.10

**Fig. 5.**
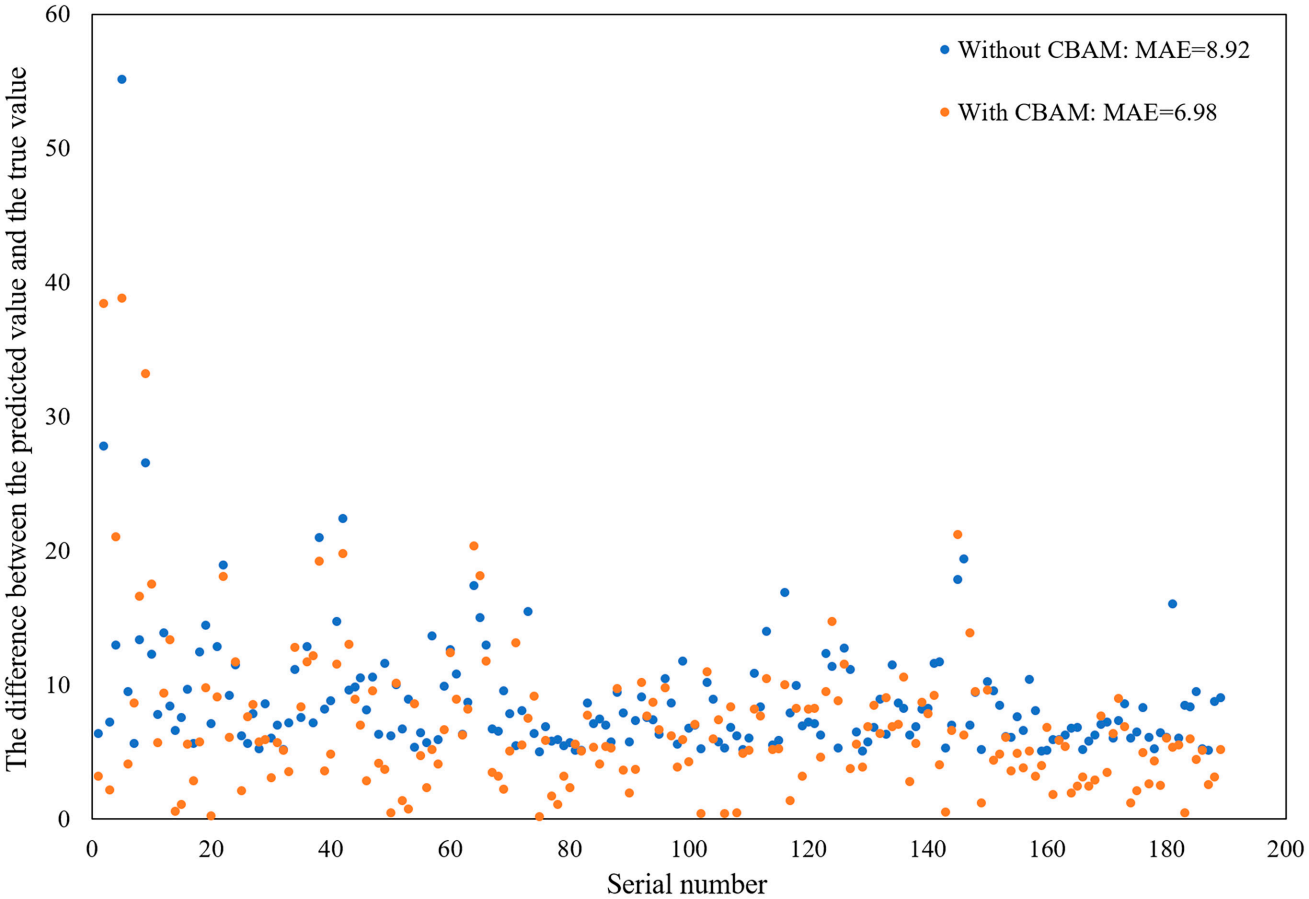
Compare the performance with and without CBAM on the images with large counting errors in the test set of the GWHD_2021 dataset.

To further illustrate the efficacy of CBAM in addressing the challenges posed by dense and complex wheat images, we select images from the test set of the GWHD_2021 dataset with larger error margins for comparison. Specifically, we use a model without CBAM to predict the absolute error (AE) of each image, and select images with AEs greater than 5. A total of 189 images, numbered from 1 to 189, are selected for this purpose. As shown in Fig. 5, the utilization of CBAM leads to a notable enhancement in the performance of the model on these challenging images, accompanied by a reduction in the MAE value from 8.92 to 6.98.

### MPM study

To confirm the effectiveness of the MPM, we vary the number of multiscale layers and size of the slices in the experiments. The smaller the slice size, the finer the features, and the more layers there are, the more spatial feature dimensions the model perceives. More specifically, we construct 1, 2, and 3 layers, in which different slice sizes are used to segment feature information in different layers, including 16 × 16, 8 × 8, and 4 × 4, resulting in 7 different structures, as summarized in Table 5. In addition, we add a finer layer to the 3-layer structure to explore the effects of additional spatial scales.

**Table 5.** The impact of different numbers of layers and slice sizes in the multiscale module on the GWHD_2020 dataset

16 × 16	8 × 8	4 × 4	2 × 2	Merging	MAE	RMSE	Number of parameters (M)	FPS
✓				✓	4.22	5.61	45.6	77.37
	✓			✓	3.61	4.66	26.9	110.81
		✓		✓	3.29	4.33	48.0	118.29
✓	✓			✓	3.36	4.51	62.8	68.31
✓		✓		✓	3.20	4.30	83.9	71.09
	✓	✓		✓	3.23	4.37	65.3	98.45
✓	✓	✓		✓	2.94	3.88	101.3	63.28
✓	✓	✓			3.09	4.04	98.8	64.89
✓	✓	✓	✓	✓	2.95	4.02	276.8	52.80

As summarized in Table 5, the MAE of slice size 4 × 4 is 8.8% lower than that of slice size 8 × 8, and 22% lower than that of slice size 16 × 16, indicating that the fineness of the features has a considerable impact on the perceptual ability of the model in the single-layer structure. In terms of the 2-layer structure, the combination of slice sizes 16 × 16 and 4 × 4 performs the best, which may be because they have a larger difference in feature sizes and thus more favorable for acquiring more different feature information. The addition of any layer with different slice sizes to the single-layer structure improves the performance. For the 3-layer structure, the MAE is reduced by 7.8% compared to the best 2-layer structure and by 10.6% compared to the best one-layer structure, confirming that multi-scaling has a positive effect on improving model performance and generalization. We also conduct ablation experiments on the merging layers after the 3 branches. First, the results demonstrate that this layer enhances the fusion of multiscale information, thereby improving the performance of the model with only a slight increase in the number of parameters. However, the 4-layer structure consumes several parameters and the performance is not improved, indicating that the limit of multiscale fusion is in the 3-layer structure. Furthermore, the 3-layer structure exhibits satisfactory speed in the inference speed test, effectively meeting the demands of real-time detection.

As summarized in Table 6, we conduct experiments on the number of layers N in the MPM to explore its impact on model performance. Notably, the performance considerably improves as the number of layers increases, with the best results achieved when *N* = 4. In particular, for the GWHD_2020 dataset, the MAE decreases from 3.08 to 2.94, whereas the RMSE decreases from 4.05 to 3.88 as *N* increases from 2 to 4. Similarly, on the GWHD_2021 dataset, the MAE decreases from 3.98 to 3.87, whereas the RMSE decreases from 5.75 to 5.60 for the same transition. However, when the number of layers is increased to 6, the performance of the model exhibits a decreasing trend and is worse than that with 2 layers. This suggests that excess layers may introduce excessive model complexity, which negatively affects the model performance in counting tasks. Hence, striking a balance in the complexity of the model when selecting layers is imperative to fully harness their accuracy in wheat ear counting.

**Table 6.** Impact of the number of layers *N* in MPM on model performance on the GWHD_2020 and GWHD_2021 datasets

Dataset	*N* = 2		*N* = 4		*N* = 6	
	MAE	RMSE	MAE	RMSE	MAE	RMSE
2020	3.08	4.05	2.94	3.88	3.19	4.16
2021	3.98	5.75	3.87	5.60	4.13	5.81

### Visualization analysis

**Fig. 6.**
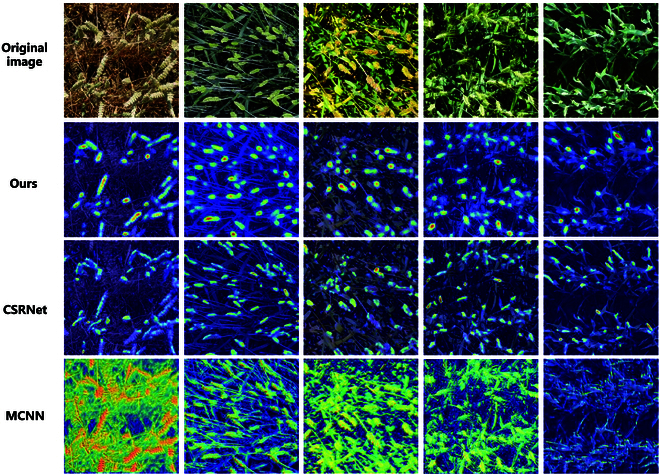
Feature visualization of the last layer from models’ backbone.

To demonstrate the superiority of the proposed method, we conduct visualization experiments using Grad-CAM [44]. Grad-CAM propagates gradients through the predicted values to obtain the gradient information for each layer. Gradient information reflects the contribution of each element to the predicted value, with larger contributions indicating that the network focuses more on them. Finally, the attention regions of the network are obtained by determining the positions of the elements with high contributions. In this section, we visualize CSNet, CSRNet [31], and MCNN [37], respectively, as shown in Fig. 6.

In specific experiments, we explore the regions of interest in the last convolutional layer of VGG16 and map them onto the original image using a heat map. In contrast, the proposed model can clearly understand the objects to be focused on even without precise location information. Compared to CSRNet and MCNN, the proposed model exhibits more stable and comprehensive attention when recognizing wheat ears of different colors, sizes, and growth stages, further proving the strong generalization and robustness of CSNet. In summary, through visualization experiments using the Grad-CAM technique, we verify the superiority of the CSNet model for wheat ear identification. We demonstrate its ability to stabilize attention to wheat ears at different characteristics and growth stages, providing important ideas for a deeper understanding and optimization of the model.

**Fig. 7.**
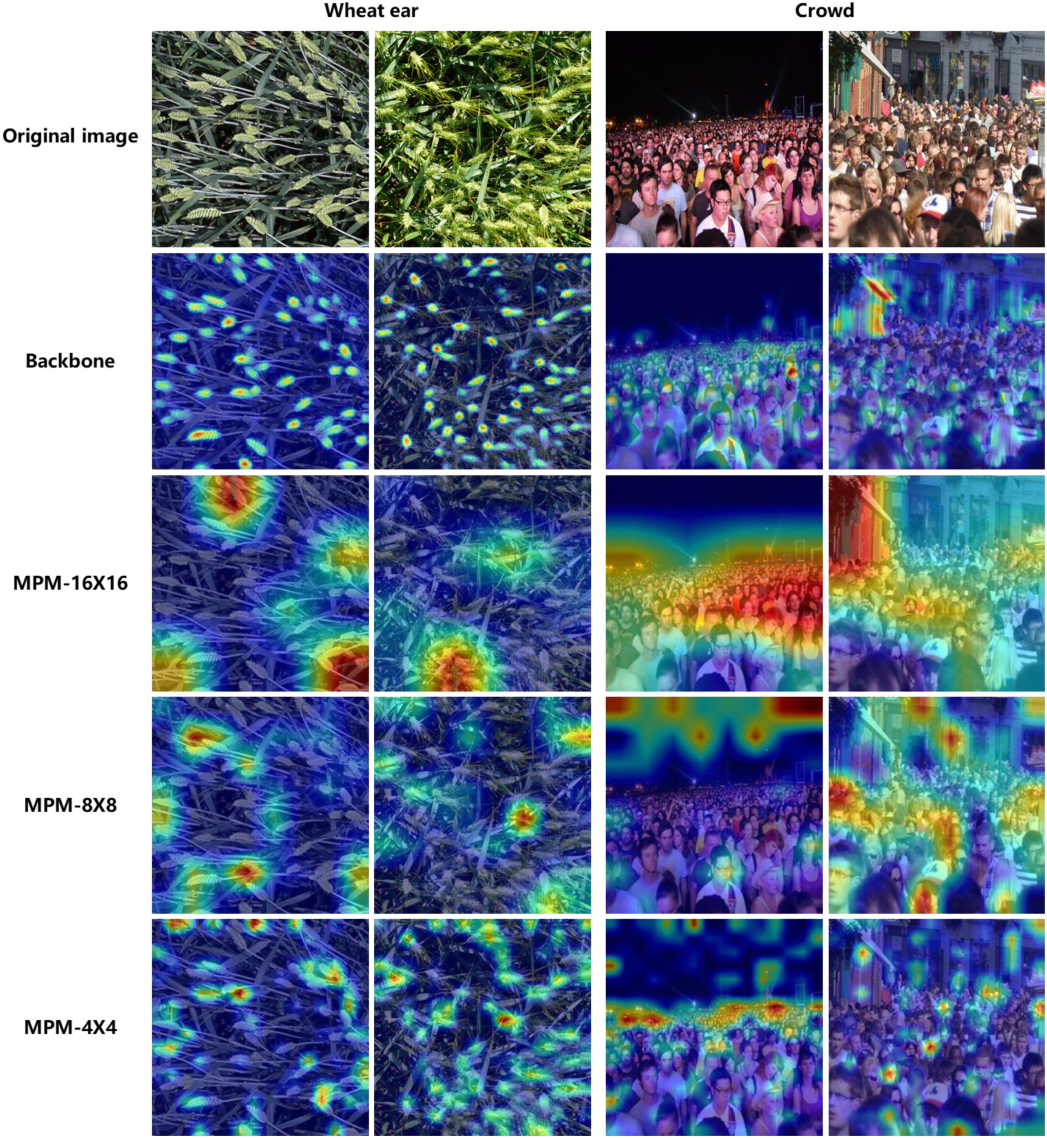
Feature visualization of 16 × 16, 8 × 8, and 4 × 4 layers in MPM and backbone layers on crowd and wheat ears.

To validate the distinctions between the MPM in CSNet and the multi-granularity MLP module in CrowdMLP [23], we conduct a visualization study on both the crowd and wheat ear datasets. In particular, because of the absence of access to the source code of the CrowdMLP model, we utilize Grad-CAM technology to generate attention heatmaps for various layers of the MPM module and the backbone layer in the wheat ear and crowd images. As shown in Fig. 7, the MPM exhibits attention features across 3 different size ranges in the wheat ear images, with larger segmentation sizes corresponding to broader attention ranges. CSNet relies on the multi-range attention mechanism in the MPM to comprehensively understand wheat ear scenes, minimize background interference, and thereby optimize its performance in wheat ear counting tasks. Because the wheat ear counting task does not pose the challenge of abrupt density variations observed in crowd counting, the design of the MPM is not specifically optimized for such challenges. Consequently, the attention of the MPM may diffuse and fail to accurately capture rapid density changes in crowd images. In contrast, CrowdMLP adopts a multi-granularity MLP module design that aims to address the rapid density variations inherent in crowd counting. The results of the visualization unequivocally demonstrate a distinct disparity in design philosophy between the MPM in CSNet and the multi-granularity MLP module in CrowdMLP. Moreover, the results demonstrate that different scales focus on different regions. Fusing them allows the model to more accurately capture a wide range of detailed and global information in the input data, thereby increasing the perceptual capabilities.

## Discussion

Counting tasks are crucial in agriculture, as counting crops (e.g., wheat, maize, and rice) can estimate growth, predict yields, and contribute to the efficiency of agricultural production. However, location-supervised methods incur high labeling costs, particularly for dense crops. To reduce the labeling cost, we propose a count-supervised model with multiscale global awareness, which achieves the best results among advanced methods. By reducing the cost and complexity of dataset creation, the proposed approach provides a more practical and cost-effective solution for automated counting in agriculture. We discuss the various aspects of CSNet in different subsections.

### Self-constructed wheat grain dataset

**Fig. 8.**
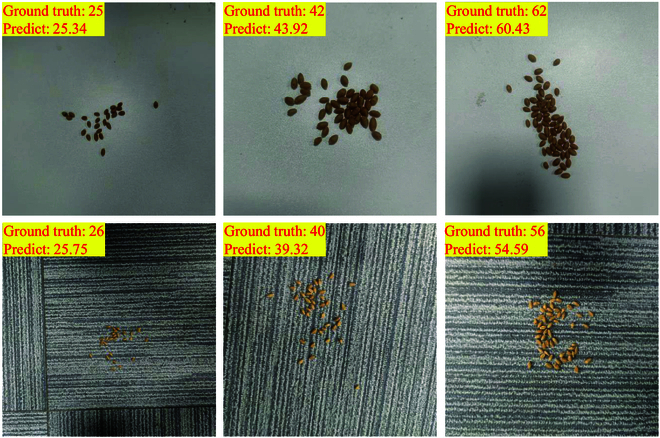
Part of the images in the self-built wheat grain dataset. This dataset contains different backgrounds and distributions.

Wheat grain count is a critical determinant of wheat yield and a key metric for evaluating crop growth and predicting production. Therefore, we employ a self-constructed wheat grain dataset to perform an extended test to illustrate the low cost and high accuracy of the proposed method. To increase the diversity and robustness of the dataset, images are captured from 2 distinct backgrounds: white and gray stripes. This deliberate variation helps train the model to adapt to a range of backgrounds and lighting conditions, thereby improving its generalization capabilities [45]. Subsequently, a predetermined number of wheat grains are randomly scattered and photographed against any given background to populate the dataset. Finally, we randomly increase or decrease the number of grains in the last shot to obtain a new image, as shown in Fig. 8, where each image is unique. In summary, because the grain count is meticulously documented during each capture, the need for extensive annotation efforts is no longer present, resulting in a low-cost dataset.

**Fig. 9.**
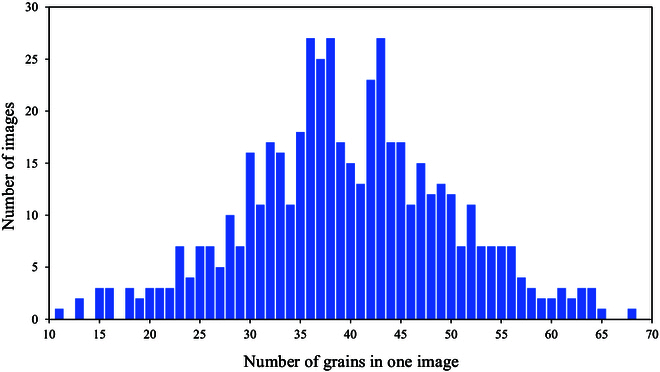
Statistical distribution of the wheat grain dataset.

In particular, we establish a wheat grain dataset with 510 images. All images contain wheat grains, except for 10 background images, which are used as negative samples. The number of grains in the image follows a Gaussian distribution, with a mean value of 40, minimum of 11, and maximum of 68. The specific distribution of the number of grains is shown in Fig. 9. We randomly select 80% of the datasets as training data and use the remainder as test data. To match the size of the wheat grains, the size of the input image is changed to 256. CSNet achieves excellent results in the experiments, with an MAE of 2.79 and an RMSE of 4.49, confirming that it can be used to predict more crops. Moreover, we conduct experiments by training on white background images and fine-tuning a small set of gray-striped background images. Test results on other gray-striped backgrounds demonstrate that fine-tuning considerably enhances the model performance, where the MAE improved from 8.30 to 3.98. The results of this experiment indicate that appropriate fine-tuning can enhance the model performance under various background conditions, thereby increasing its usefulness and robustness in real-world applications.

### Exploration of MPM

Multiscale techniques have been widely adopted and proven to be effective in computer vision for capturing different features in images, thereby enhancing the understanding and generalization capabilities of a model across different objects or scenes [46–48]. Given the potential variations in wheat fields, including different growth stages, varieties, and wheat ear densities, we introduce multiscale techniques to improve the adaptability of the proposed counting model to complex scenes. Initially, we adopt a pyramid structure commonly used in the visual domain to generate multiple feature maps of different sizes. We attempt to slice each feature map into equally sized slices to capture information at different spatial scales. Nevertheless, the experiments in this study reveal that this structure impedes the ability of the network to perceive wheat ear features, possibly because of the misalignment of semantic information across multiple feature maps. To ensure consistent semantic information, we slice a single feature map into segments of different sizes to capture the information at multiple spatial scales.

Furthermore, to address the challenge of perceiving objects without location information, selecting a structure with a global receptive field is crucial. In contrast to Transformers, the structure of the MLP-Mixer does not rely on self-attention mechanisms. This characteristic enables the MLP-Mixer to train and generalize more effectively with limited data. Considering the relatively straightforward nature of the wheat ear counting task and the relatively small dataset, which does not require complex context understanding or long-range dependency modeling, the concise structure of the MLP-Mixer proves to be more appropriate. The absence of self-attention mechanisms makes the model easier to train, and it exhibits superior performance in resource-constrained scenarios, making it a more appropriate choice. However, if the MLP-Mixer is used directly for counting, it will achieve unsatisfactory results in wheat counting, and its experimental result on the GWHD_2020 dataset exhibits an MAE of 12.83.

In crowd images, individuals at varying distances exhibit substantial differences in size, resulting in notable density variations across different positions in the image. The multi-granularity MLP module in CrowdMLP is specifically designed to address rapid density changes in crowd-counting challenges. This module effectively captures and integrates the semantic information from different granularities, thereby improving the adaptability of the model to variations in crowd density. Compared to the proposed MPM, the design focus of the CrowdMLP module is on density changes within crowds. Conversely, the proposed module addresses different growth stages and varieties in wheat scenes by extracting information at different spatial scales. The distinct design philosophies of the 2 modules enable each to excel in their respective scenarios, exhibiting optimal performance.

### Application prospects

Counting is a crucial task in the field of agriculture that provides accurate data support to farmers and aids scientific agricultural management and production decisions [49]. With the advancements in computer vision, agricultural counting has gradually become more automated and intelligent [50]. However, the high cost of creating datasets has emerged as a bottleneck, hindering the widespread adoption of this technology and the struggle to meet the diverse counting requirements of agriculture. Therefore, the proposed method aims to reduce the cost of dataset creation, thereby enabling low-cost automated counting. In contrast, several agricultural quantity assessments are currently performed manually, and a small count-supervised dataset can be obtained by additionally taking images. Capturing images from various angles in a single region allows label reuse and reduces annotation costs. Furthermore, for plants grown in regional settings (e.g., grapes and tomatoes), a camera can be used to capture panning shots. In such instances, fruits of the same cluster appearing in different images must be counted only once, thereby reducing the occurrence of double counting. For neatly planted crops, quantitative information can be quickly obtained by manually recording the number of rows and columns. However, for densely planted or widely planted crops, manually counting the number of rows and columns can be tedious. CSNet is an effective solution for automating the counting process while minimizing the cost of labeling.

## Conclusion

In this study, we propose a novel method for the accurate and efficient counting of wheat ears using count-supervised methods. First, we design a multiscale model with global perception to utilize counting information to learn the attention graph between the wheat ear features, which includes a backbone, CBAM [25], MPM, and CM. The backbone and CBAM are primarily used to extract wheat ear features and reduce interference from complex backgrounds. The MPM learns the relationship graph between wheat ear features from a multidimensional space, which is conducive to identifying the intrinsic connection between wheat ear features and counting information. To validate the proposed approach, experiments are conducted using the global wheat ear head detection dataset [26,27]. In comparative experiments, we compare box-supervised and point-supervised approaches and achieve superior results. The effectiveness of the MPM is validated in ablation experiments and demonstrated through a visual analysis of the attention maps of CSNet. Finally, we establish a wheat grain dataset for experiments, which is applied to evaluate the time cost required for count- and position-level annotations, and to verify the robustness and generality of the proposed method. The results demonstrate that the proposed method not only reduces the cost of creating datasets but also exhibits excellent counting performance.

## Data Availability

Data are available at https://github.com/GZU-SAMLab/CSNethttps://github.com/GZU-SAMLab/CSNet.
